# Body Mass Index Lacks Predictive Influence on Perioperative, Short-Term Follow-Up, and Patient-Reported Outcomes from Holmium Laser Enucleation of the Prostate

**DOI:** 10.3390/jpm16040225

**Published:** 2026-04-18

**Authors:** Jack T. Peterson, Jenny N. Guo, Amir Patel, Nabila Khondakar, Perry Xu, Amy E. Krambeck

**Affiliations:** 1 Department of Urology, Northwestern University Feinberg School of Medicine, Chicago, IL 60611, USA; 2 Department of Urology, Oregon Health and Science University, Portland, OR 97239, USA

**Keywords:** Holmium laser enucleation of the prostate, benign prostatic hyperplasia, body mass index, obesity, lower urinary tract symptoms, retrospective cohort study

## Abstract

**Background/Objectives**: Obesity has been associated with the development and severity of benign prostatic hyperplasia (BPH), yet its influence on outcomes following definitive surgical management, like holmium laser enucleation of the prostate (HoLEP), remains unclear. Furthermore, gradation of body mass index (BMI) severity has yet to discern personalized outcome stratification. We evaluated BMI’s influence on perioperative, immediate, short-term follow-up, and patient-reported outcomes for HoLEP patients. **Methods**: We performed a retrospective review of a prospectively maintained database of patients undergoing HoLEP for BPH at a single institution between January 2021 and August 2025. Outcomes included operative characteristics, post-operative complications, and validated symptom score changes. Analyses treated BMI as both a continuous and categorical variable. Multivariable linear and logistic regression models adjusted for common colinear confounders. **Results**: Among 1445 patients, BMI was not associated with most immediate, three-month, or patient-reported outcomes. Surgical complications were low across all BMI categories, and post-operative reported outcomes indicating high success rate for HoLEP. Higher BMI correlated with a modest increase in enucleation time (β = 0.197; *p* = 0.0132), increased odds of dysuria (OR = 1.084; *p* < 0.001), and change in American Urological Association Symptom Score (β = 0.211; *p* = 0.0334). All other operative metrics, complication rates, continence outcomes, and symptom scores (17 other total) were independent of BMI. **Conclusions**: After adjustment for relevant confounders, BMI does not meaningfully predict surgical safety, functional recovery, or patient-reported benefit following HoLEP. BMI alone should not influence candidacy or risk stratification for HoLEP in patients with BPH, instead favoring personalized, risk-stratified approaches.

## 1. Introduction

Obesity is a major risk factor in the development of benign prostatic hyperplasia (BPH) and associated lower urinary tract symptoms (LUTSs), a highly prevalent condition that affects over 80% of men 70 years old and older [1,2]. Studies have shown that elevated body mass index (BMI), a common calculation from height and mass used to classify body weight categories, increases likelihood of more intense and rapidly onsetting LUTS [3]. A variety of etiological explanations exist, including elevated intra-abdominal pressures increasing bladder pressure, an endocrine-hypogonadal dysfunction, and increased sympathetic activation and inflammation [3]. Since the global obesity epidemic, more patients are presenting with concomitant LUTS and elevated BMI. The literature suggests that obese patients are less likely to experience significant benefits from pharmaceutical therapy alone, and with further progression of symptoms, obese patients are often more inclined to receive surgical intervention for definitive treatment [4].

Patients with obesity who receive surgical treatment options have nuanced background profiles compared to other patients with BPH. In a study by Chen et al., patients with obesity undergoing Greenlight photo-vaporization of the prostate (PVP) were found to be younger with larger transition zones and total prostate volume [5]. However, this was not correlated to discrepancies in either surgical parameters or complications. When reviewing patients who received prostatectomies via either transhow resection of the prostate (TURP) or Greenlight laser selective PVP, surgical complications, such as retention of urine, bladder stones and diverticula formation, were not significantly different for overweight and obese patients [6]. Similar observations were noted for thulium laser vapoenucleation [7]. These studies, however, notably did not control for several other comorbidities which are also significantly more prevalent in patients with obesity. Thus, the many associated patient characteristics in patients with obesity and LUTS must be sufficiently accounted for when assessing BMI’s influence. Obesity commonly coexists with metabolic syndromes, particularly diabetes and neurologic diseases, which independently influence bladder function, LUTS, and recovery. Prior studies therefore often capture combined effects rather than BMI alone. Excluding these conditions can isolate BMI’s influence, enabling more precise, personalized risk stratification and interpretation.

Holmium laser enucleation of the prostate (HoLEP) is an American Urological Association (AUA)-recommended treatment option for LUTS and BPH, independent of prostate and gland size [8]. It has been employed in patients with elevated BMIs, who also typically have larger prostate volumes [9]. Obesity significance testing with post-operative complications has proven difficult, due to HoLEP’s low complication rate, but obesity has been correlated to longer operation times in prior studies [10]. One complication of note was higher incidence of open conversion for patients with elevated BMIs receiving HoLEP due to their body habitus and difficulty with scope manipulation [11]. However, the current body of literature has failed to control for colinear patient characteristics and has been performed on too small of a sample pool to aptly capture surgical complications and adverse events. We sought to address this gap by investigating the influence of BMI on an array of surgical characteristics and outcomes related to HoLEP.

## 2. Materials and Methods

Following IRB approval, we conducted a retrospective review of a prospectively maintained database of patients undergoing HoLEP surgery for BPH at our institution between January 2021 and August 2025. All HoLEP procedures were performed by one of two surgeons using high-powered pulse-modulated holmium laser technology. Exclusion criteria included patients with history of diabetes, neurological disease, urinary tract infections, prostatitis, urethral stricture, or radiation therapy. Diabetes and neurologic diseases have been previously correlated with worse surgical outcomes following HoLEP, and, given their higher prevalence in obese patients, were excluded (*n* = 342) [12]. Patients with intra-operative complications or receiving hemi-HoLEP (partial/half gland removal) totaled *n* = 81 and *n* = 8 respectively and were also excluded. Surgeries with intra-operative complications were removed given that this investigation studied outcome courses, all of which would be influenced by the complication itself instead of BMI alone. Post-operative survey responses, patient demographics, baseline health status, symptom scores, and urinary function; operative characteristics; immediate post-operative details; functional outcomes; and adverse event incidences were all collected for each participant in our study. Patients missing greater than 25% of this information were also excluded.

Analysis was performed with a set of six confounding variables. Patient’s age, pre-operative prostate volume, prostate-specific antigen level, American Society of Anesthesiologists (ASA) physical status classification, occurrence of a prior BPH surgery, and history of anticoagulation use at time of surgery. These potential confounders are all indicated to potentially influence peri- and post-operative characteristics and outcomes, and all could have been correlated to a patient’s BMI. To analyze patient BMI as the independent variable, while also accounting for clinical relevance of standard BMI categories, two series of statistics were performed in parallel. First, BMI was treated as a continuous variable. Subsequently, a BMI category of underweight, normal weight, overweight, and obese was assigned to each patient: buckets were under 18.5, 18.5 to 24.9, 25 to 29.9, and 30 or greater kg/m^2^, respectively. For categorical analyses, BMI was modeled using indicator (dummy) variables with the healthy BMI range as the reference category, allowing estimation of category-specific effects while preserving within-group variability. The underweight group was excluded from categorical analysis due to insufficient sample size (*n* = 2) for stable inference.

For continuous outcome variables, linear regressions were fit, accounting for confounders, between patient BMI and the outcome, and β values for the regression coefficient were recorded. For categorical variables, such as for the occurrence of an event, logistic regressions, also accounting for confounding variables, were employed, with odds ratios (ORs) calculated. For all outcome analyses, sample size varied by endpoint based on data availability. General statistics utilized all available data for each variable. In contrast, multivariable analyses were conducted using complete-case samples, such that the analytic denominator for each model includes only patients with non-missing BMI, covariates, and the outcome of interest. Accordingly, sample sizes differ across outcomes and models. For both β and OR, 95% confidence intervals (CIs) and *p*-values were derived. All analyses were conducted using Python 3.0 (Python Software Foundation, Wilmington, DE, USA). Statistical significance was defined as *p* < 0.05. Due to the exploratory nature of this work, *p*-value adjustments, such as a Bonferroni correction, were not employed.

## 3. Results

**Figure 1:**
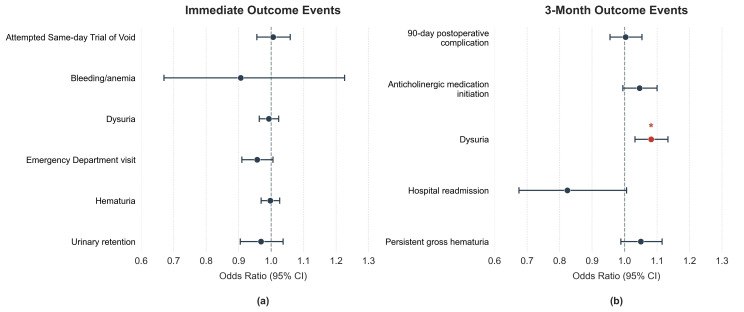
Forest Plot of odds ratio for outcome incidences based on BMI as a continuous value: (**a**) Immediate outcome events; (**b**) 3-month outcome events. Error bars depict 95% confidence intervals. Significance indicated by * and red color. CI, Confidence interval.

A total of 1445 patients who underwent HoLEP were included in this study (Table 1). Patients were on average 70.2 ± 8.0 years old, had an average prostate size of 113.0 ± 63.7 g and prostate-specific antigen (PSA) of 5.9 ± 7.6 ng/mL. A history of prior BPH surgery was present in 14.2% of patients and 13.8% were on anticoagulation at the time of surgery. A total of 70 patients were both excluded from the study due to intra-operative complications and had reported BMIs. Their respective BMI distribution was similar to that of the study cohort’s following a 2-sample Kolmogorov–Smirnov test (intra-operative complication cohort: 28.1 ± 4.1 kg/m^2^; study cohort: 27.6 ± 4.6 kg/m^2^; *p* = 0.405). Overall, only three of the twenty performed statistical tests crossed our predetermined α of 0.05—operative enucleation time, dysuria at 3 months post-operatively, and American Urological Association Symptom score (AUASS) change (Figure 1). Statistical significance and overall trends were largely mirrored between testing by BMI category and when viewing BMI as a continuous variable, thus the table below references only the latter (the former can be viewed in detail comparing healthy BMI range patients to either overweight or obese BMI range patients in Appendix A tables [Table A1, Table A2, Table A3, Table A4, Table A5 and Table A6]).

### 3.1. Immediate Post-Surgery Characteristics

Table 2 summarizes immediate post-operative outcomes and their associations with BMI; almost none of these outcomes depicted a significant relationship with BMI. Among continuous measures, BMI was not associated with operating room time, morcellation time, total laser energy delivered, or prostate specimen weight. Enucleation time was the sole exception, with a modest increase with increasing BMI (β 0.197; *p* = 0.0132). Binary post-operative events likewise demonstrated no significant associations, with odds ratios near unity and wide confidence intervals (*p* > 0.05 for all). Overall, immediate post-operative outcomes after HoLEP appeared largely independent of BMI, except for a small but statistically significant increase in enucleation time.

### 3.2. 3-Month Post-Operative Characteristics

Table 3 details patients’ three-month post-operative outcomes and correlations to their BMI. Most outcomes again showed no statistically significant relationship with BMI. BMI lacked a significant correlation to 90-day post-operative complications or hospital readmissions. Dysuria was the only statistically significant three-month outcome; higher BMI correlated to increased odds of occurrence (11.6%; OR 1.084 [1.034–1.136]; *p* < 0.001). However, in aggregate, three-month post-operative outcomes, similarly to immediate outcomes, were largely independent of BMI.

### 3.3. Survey Score Changes

Changes in AUA symptom score (AUASS) and Michigan Incontinence Symptom Index (M-ISI) scores are documented in Table 4. These scores generally decreased following HoLEP, with AUASS dropping the most (decrease of 11.19 ± 9.13; β 0.211; *p* = 0.0334). This was the sole change significantly correlated to BMI.

## 4. Discussion

Results from our investigation suggest that BMI is not an independent predictor of perioperative, short-term follow-up, or patient-reported outcomes following HoLEP. This is following statistical control of clinically relevant confounders. This work mirrors findings from other prostate resection surgical techniques for BPH. In the Greenlight PVP literature, Chen et al. and Pierce et al. studied similar surgical outcomes, also noting BMI’s lack of influence [5,13]. Previous characterizations of obesity’s deleterious effect in BPH treatment have centered on increased waist circumference or BMI in the setting of metabolic syndrome [14]. These studies proceed to associate obesity with persistent storage LUTS following open prostatectomies and TURP; however, this contextually skews results, due to other metabolic syndrome comorbidities like diabetes or hypertension. Alternatively, we chose confounding variables based on their collinearity with obesity itself, to better segregate BMI’s effect. Further, we excluded neurological diseases and diabetes mellitus, often considered a large confounding comorbid condition in urology studies involving obesity [15]. While this limits the generalizability of the true clinical effect of an elevated BMI, omitting common sequalae, the purpose of this study was to investigate the influence of the BMI metric in isolation at risk stratification, necessitating removal of as much confounding as possible.

Three of our outcome variables were significantly correlated to BMI following confounder control: enucleation time, dysuria, and AUASS change. While we elected not to perform *p*-value adjustments due to this investigation’s exploratory nature, given we performed 20 comparisons, this is an inherent limitation. Particularly for AUASS change, which had a *p*-value of 0.0334, more caution is required when generalizing results. The significant difference in enucleation time is likely a reflection of larger prostates or more challenging tissue planes, which would result in longer time for enucleation. This has been similarly shown in the literature where Tamalunas et al. noted a twelve-minute increase in enucleation time from 31 to 43 min in patients with obesity (*p* < 0.01) [10]. Chen et al. similarly noted larger prostate sizes (*p* < 0.001) and transition zone volumes (*p* = 0.017) with BMI for patients receiving Greenlight PVP [5]. While our result was also significant (*p* = 0.0132), this would likely not remain following *p*-value adjustment, suggesting a small effect size or a side effect of our large sample cohort and high number of significance tests performed.

Dysuria was also significantly more common as patient BMI category increased (OR: 1.084; *p*-value < 0.001). This may be a factor of the patient’s BPH itself, where obesity and an elevated BMI have previously been correlated to increased LUTSs, including dysuria specifically [16]. In Greenlight PVP, Pierce et al. found that obese patients required higher energy utilization compared to normal BMI patients [13]. Higher energy utilization can lead to increased mucosal irritation and transient dysuria. In our study, we did not find high total energy utilization with increased BMI. However, other mechanistic explanations include delayed epithelial healing in metabolic disease, altered nociceptive signaling, and undiagnosed prediabetes/diabetes. Diabetes mellitus has been shown to significantly correlate to dysuria symptoms in men, due to diabetic autonomic neuropathy causing genitourinary disturbances [17,18]. While we excluded any patient with a diabetes diagnosis, given that it will be more prevalent in patients with obesity, this cohort is more likely to be prediabetic or have undiagnosed diabetes. This could explain the higher dysuria rate with increasing BMI category. Baseline hemoglobin A1c (HbA1c) was not available for these patients, preventing any subsequent analysis on its correlation. Lacking this or any other indication of prediabetes or uncontrolled glucose levels limits substantiating this assumption. While our study did not directly quantify prediabetes or HbA1c, a prior HoLEP study found no differences in outcomes after stratifying patients based on HbA1c levels [12].

It is worth noting that larger body habitus can make HoLEP technically challenging to perform, causing some surgeons to abort cases or convert to TURP and/or open surgery [19]. In our cohort, we were able to successfully perform HoLEPs on patients with BMIs up to 49.66 kg/m^2^ with zero conversions or case abortions. This success may be due, in part, to an adaptive surgical strategy that modifies our traditional “bottom-up” approach as needed. Specifically, if we are unable to enter the bladder neck initially, we create a posterior incision distally starting at the verumontanum and move proximally toward the bladder neck. The anterior plane of the prostate is generally shorter than the posterior plane, allowing for easier entry into the bladder. Once bladder entry is obtained through the anterior plane, the lateral margins of the bladder neck are opened dissected in a top-down approach. The high-powered holmium laser offers excellent hemostasis which minimizes bleeding risks that are otherwise amplified in obesity, making HoLEP an excellent choice for obese patients with LUTS [20,21]. The low complication rate of HoLEP may be a limitation in itself, due to sparse events limiting reliability of logistic regressions for subgroups. This is countered by our large sample size (*n* = 1445 patients), but generalizability concerns persist given how rare several of these events are.

Our study consists of several limitations due to its retrospective single-center nature. This includes establishing only correlations instead of causation, a low frequency of adverse events limiting the power of significance tests, and a lack of randomization. Although patients depicted a wide distribution of BMI values, other demographic variables may be more homogeneous given that they all received care from the same institution. These limitations are countered by our large overall sample size of 1445 patients, our uniform procedural, data collection, and statistical techniques, and our robust confounder control. Further, studying multiple outcomes from different temporal domains offered a broader, more inclusive depiction of surgical outcomes from HoLEP.

Our investigation suggests that patient BMI does not meaningfully influence surgical safety, functional recovery, or patient-perceived benefit following HoLEP, and thus should not be used as a sole determinant in clinical decision-making. Albeit via a retrospective investigation with clinically relevant exclusion criteria, this study suggests that specifically BMI may lack clinical utility. Given the lack of statistical correlation, patient BMI alone does not appear to be a deterrent for surgical operation, nor could it act as a surrogate for surgical risk. Categorizing patients as underweight, normal weight, overweight, or obese, albeit contributory to developing BPH in the first place, fails to establish insight into their surgical outcomes. Other patient demographics or comorbidities may contribute to a more complete characterization of HoLEP efficacy, but this research dissuades reliance on BMI alone. Suitability of this surgical procedure remains reliant on the shared decision-making and discretion of the urologist and patient.

## 5. Conclusions

BMI, both when utilized as a continuous numerical calculation and as a delineator for weight categories, fails to significantly influence most surgical outcomes and characteristics following HoLEP when controlling for other prevalent confounding variables. This counters previous dogmas on the risk factor of obesity or elevated BMI values alone for complications and adverse outcomes and instead insinuates the necessity to reframe clinical judgment unto other aspects of the patient’s health. While this does not comment on BMI’s influence on the occurrence of BPH itself, the significance of other prevalent comorbidities should be further investigated to similarly assess their impact on surgical outcomes following HoLEP. This supports a proposal for individualized risk stratification, composed of patients’ entire clinical profile, when identifying candidacy for and benefit from HoLEP.

## Figures and Tables

**Table 1:** Demographics and intra-operative characteristics for the cohort and by BMI category.

Characteristic	Overall	Underweight	Healthy	Overweight	Obese
N	1445	2	456	622	365
Baseline Characteristics					
Age (years)	70.2 ± 8.0 (1313)	69.0 ± 4.8 (2)	70.7 ± 8.3 (415)	70.3 ± 7.8 (564)	69.6 ± 7.9 (332)
BMI (kg/m^2^)	27.6 ± 4.6 (1445)	18.2 ± 0.1 (2)	23.0 ± 1.4 (456)	27.3 ± 1.4 (622)	33.7 ± 3.6 (365)
Prostate size (cc)	113.0 ± 63.7 (1186)	79.5 ± 16.3 (2)	107.3 ± 60.1 (377)	111.7 ± 63.1 (501)	122.4 ± 68.3 (306)
PSA (ng/mL)	5.9 ± 7.6 (1322)	38.9 ± 0 (1)	6.2 ± 8.4 (412)	6.1 ± 7.7 (568)	5.2 ± 5.9 (341)
AUASS symptom score	19.4 ± 7.5 (761)	—	18.8 ± 7.6 (220)	19.9 ± 7.6 (335)	19.1 ± 7.3 (206)
AUASS quality of life score	4.1 ± 1.3 (576)	—	4.1 ± 1.3 (169)	4.1 ± 1.3 (247)	4.2 ± 1.3 (160)
M-ISI severity	5.4 ± 5.5 (619)	—	4.8 ± 5.5 (170)	5.6 ± 5.3 (274)	5.9 ± 5.8 (175)
M-ISI bother	1.8 ± 2.3 (625)	—	1.6 ± 2.1 (171)	1.9 ± 2.3 (276)	1.8 ± 2.4 (178)
Intra-operative Characteristics					
Operating room time (minutes)	59.3 ± 29.2 (1378)	50.0 ± 15.6 (2)	57.9 ± 29.9 (433)	58.3 ± 26.4 (601)	62.8 ± 32.7 (342)
Enucleation time (minutes)	29.6 ± 14.0 (1423)	28.0 ± 15.6 (2)	28.8 ± 12.5 (447)	29.0 ± 12.5 (616)	31.5 ± 17.8 (358)
Morcellation time (minutes)	8.7 ± 8.8 (1421)	4.0 ± 0.0 (2)	8.2 ± 7.8 (446)	8.5 ± 8.7 (616)	9.7 ± 10.0 (357)
Total energy (kJ)	135.6 ± 65.6 (1375)	110.0 ± 4.5 (2)	133.4 ± 68.1 (431)	134.1 ± 64.6 (597)	141.1 ± 64.4 (345)
Prostate specimen weight (grams)	73.2 ± 57.2 (1386)	41.0 ± 18.4 (2)	70.2 ± 54.7 (435)	71.6 ± 56.4 (601)	79.9 ± 61.3 (348)
Immediate Post-operative Characteristics					
Attempted same-day trial of void	1099/1274 (76.1%)	2/2 (100.0%)	334/400 (83.5%)	485/549 (88.3%)	278/323 (86.1%)
Hematuria	559/1324 (42.2%)	1/1 (100.0%)	187/419 (44.6%)	236/578 (40.8%)	135/326 (41.4%)
Dysuria	444/1304 (34.0%)	1/1 (100.0%)	132/410 (32.2%)	207/572 (36.2%)	104/321 (32.4%)
Urinary tract infection	7/1023 (0.7%)	0/1 (0.0%)	2/324 (0.6%)	4/444 (0.9%)	1/254 (0.4%)
Emergency department visit	140/1313 (10.7%)	0/2 (0.0%)	53/412 (12.9%)	56/576 (9.7%)	31/323 (9.6%)
Urinary retention	82/396 (20.7%)	—	36/129 (27.9%)	27/176 (15.3%)	19/91 (20.9%)
3-month Post-operative Characteristics					
90-day complication	121/831 (14.6%)	—	42/255 (16.5%)	41/364 (11.3%)	38/212 (17.9%)
Readmission	15/98 (15.3%)	—	10/34 (29.4%)	4/32 (12.5%)	1/32 (3.1%)
Persistent gross hematuria	65/959 (6.8%)	—	16/290 (5.5%)	26/426 (6.1%)	23/243 (9.5%)
Dysuria	108/960 (11.2%)	—	20/292 (6.8%)	49/425 (11.5%)	39/243 (16.0%)
Anticholinergic initiation	104/717 (14.5%)	—	25/223 (11.2%)	48/317 (15.1%)	31/177 (17.5%)

Abbreviations: BMI, body mass index; PSA, prostate-specific antigen; AUASS, American Urological Symptom Score; M-ISI, Male Incontinence Symptom Index. Continuous variables are presented as mean ± standard deviation (N) where N reflects the number of patients with available data. Categorical variables are presented as n/N (percentage). BMI categories were defined as Underweight (<18.5 kg/m^2^), Healthy (18.5–24.9 kg/m^2^), Overweight (25.0–29.9 kg/m^2^), and Obese (≥30.0 kg/m^2^). Age was calculated at the time of surgery.

**Table 2:** Immediate outcome events and characteristics with their correlation to BMI as a continuous variable.

Outcome	β/Odds Ratio (95% Confidence Interval)	*p*-Value
Attempted Same-day Trial of Void	OR: 1.005 (0.955, 1.058)	0.843
Operating Room time	β: 0.244 (−0.043, 0.530)	0.0951
Enucleation time	β: 0.197 (0.041, 0.352)	0.0132 *
Morcellation time	β: 0.004 (−0.076, 0.083)	0.930
Total laser energy delivered	β: 0.223 (−0.367, 0.812)	0.459
Prostate specimen weight	β: 0.096 (−0.329, 0.521)	0.657
Hematuria	OR: 0.997 (0.969, 1.026)	0.857
Dysuria	OR: 0.994 (0.964, 1.024)	0.679
Urinary Tract Infection	OR: 0.988 (0.814, 1.199)	0.900
Emergency Department visit	OR: 0.956 (0.909, 1.005)	0.0794
Urinary retention	OR: 0.967 (0.904, 1.036)	0.340

Abbreviations: BMI, body mass index; *, *p* < 0.05.

**Table 3:** Three-month outcome events with their correlation to BMI as a continuous variable.

Outcome	β/Odds Ratio (95% Confidence Interval)	*p*-Value
90-day post-operative complication	OR: 1.003 (0.955, 1.053)	0.919
Hospital readmission	OR: 0.824 (0.675, 1.006)	0.0573
Persistent gross hematuria	OR: 1.054 (0.993, 1.119)	0.0854
Dysuria	OR: 1.084 (1.034, 1.136)	<0.001 *
Anticholinergic medication initiation	OR: 1.047 (0.995, 1.100)	0.0749

Abbreviations: BMI, body mass index; *, *p* < 0.05.

**Table 4:** Change in survey response scores with their correlation to BMI as a continuous variable.

Outcome	β/Odds Ratio (95% Confidence Interval)	*p*-Value
AUASS change	β: 0.211 (0.017, 0.406)	0.0334 *
AUASS Quality of Life change	β: 0.030 (−0.020, 0.079)	0.239
M-ISI severity score change	β: 0.047 (−0.090, 0.184)	0.502
M-ISI bother score change	β: 0.015 (−0.052, 0.083)	0.658

Abbreviations: BMI, body mass index; AUASS, American Urological Symptom Score; M-ISI, Male Incontinence Symptom Index; *, *p* < 0.05.

## Data Availability

Data availability is not applicable to this article, given that no new data was created or analyzed.

## Abbreviations

The following abbreviations are used in this manuscript:

BPH	Benign Prostatic Hyperplasia
HoLEP	Holmium Laser Enucleation of the Prostate
BMI	Body Mass Index
LUTS	Lower Urinary Tract Symptoms
PVP	Photo-Vaporization of the Prostate
TURP	Transurethral Resection of the Prostate
AUA	American Urological Association
ASA	American Society of Anesthesiologists
PSA	Prostate-Specific Antigen
HbA1c	Hemoglobin A1c
AUASS	American Urological Association Symptom Score
IPSS	International Prostate Symptom Score
M-ISI	Michigan Incontinence Symptom Index
QOL	Quality of Life
IRB	Institutional Review Board
TOV	Trial of Void
OR	Odds Ratio
CI	Confidence Interval

## Appendix A

**Table A1:** Immediate outcome events and characteristics compared between healthy BMI range and overweight BMI range patients.

Outcome	β/Odds Ratio (95% Confidence Interval)	*p*-Value
Attempted Same-day Trial of Void	OR: 1.269 (0.734, 2.195)	0.394
Operating Room time	β: 0.789 (−2.115, 3.692)	0.594
Enucleation time	β: 0.511 (−1.131, 2.154)	0.542
Morcellation time	β: −0.414 (−1.254, 0.426)	0.334
Total laser energy delivered	β: 0.635 (−5.446, 6.716)	0.838
Prostate specimen weight	β: −0.986 (−5.489, 3.517)	0.668
Hematuria	OR: 0.900 (0.670, 1.209)	0.484
Dysuria	OR: 1.214 (0.887, 1.662)	0.225
Urinary Tract Infection	OR: 3.467 (0.363, 33.098)	0.280
Emergency Department visit	OR: 0.704 (0.438, 1.133)	0.148
Urinary retention	OR: 0.517 (0.265, 1.009)	0.054

Abbreviations: BMI, body mass index.

**Table A2:** Immediate outcome events and characteristics compared between healthy BMI range and obese BMI range patients.

Outcome	β/Odds Ratio (95% Confidence Interval)	*p*-Value
Attempted Same-day Trial of Void	OR: 1.071 (0.586, 1.958)	0.822
Operating Room time	β: 1.650 (−1.726, 5.027)	0.338
Enucleation time	β: 1.943 (0.039, 3.848)	0.0455 *
Morcellation time	β: −0.078 (−1.053, 0.897)	0.875
Total laser energy delivered	β: 1.979 (−5.081, 9.040)	0.582
Prostate specimen weight	β: 2.416 (−2.801, 7.634)	0.364
Hematuria	OR: 0.900 (0.637, 1.270)	0.547
Dysuria	OR: 1.051 (0.729, 1.518)	0.789
Urinary Tract Infection	OR: 1.536 (0.086, 27.449)	0.771
Emergency Department visit	OR: 0.690 (0.391, 1.215)	0.198
Urinary retention	OR: 0.725 (0.338, 1.557)	0.410

Abbreviations: BMI, body mass index; *, *p* < 0.05.

**Table A3:** Three-month outcome events compared between healthy BMI range and overweight BMI range patients.

Outcome	Odds Ratio (95% Confidence Interval)	*p*-Value
90-day post-operative complication	0.718 (0.423, 1.218)	0.219
Hospital readmission	0.301 (0.060, 1.520)	0.146
Persistent gross hematuria	1.096 (0.495, 2.426)	0.821
Dysuria	1.740 (0.926, 3.272)	0.085
Anticholinergic medication initiation	1.393 (0.783, 2.477)	0.260

Abbreviations: BMI, body mass index.

**Table A4:** Three-month outcome events compared between healthy BMI range and obese BMI range patients.

Outcome	Odds Ratio (95% Confidence Interval)	*p*-Value
90-day post-operative complication	1.220 (0.691, 2.155)	0.493
Hospital readmission	0.112 (0.011, 1.099)	0.060
Persistent gross hematuria	2.177 (0.975, 4.858)	0.058
Dysuria	3.323 (1.731, 6.380)	<0.001 *
Anticholinergic medication initiation	1.540 (0.808, 2.934)	0.190

Abbreviations: BMI, body mass index; *, *p* < 0.05.

**Table A5:** Change in survey response scores compared between healthy BMI range and overweight BMI range patients.

Outcome	β (95% Confidence Interval)	*p*-Value
AUASS change	−1.009 (−3.142, 1.124)	0.354
AUASS Quality of Life change	0.050 (−0.480, 0.581)	0.852
M-ISI severity score change	0.747 (−0.914, 2.407)	0.378
M-ISI bother score change	0.136 (−0.680, 0.952)	0.745

Abbreviations: BMI, body mass index; AUASS, American Urological Symptom Score; M-ISI, Male Incontinence Symptom Index.

**Table A6:** Change in survey response scores compared between healthy BMI range and obese BMI range patients.

Outcome	β (95% Confidence Interval)	*p*-Value
AUASS change	2.107 (−0.311, 4.526)	0.0877
AUASS Quality of Life change	0.455 (−0.148, 1.058)	0.139
M-ISI severity score change	0.583 (−1.255, 2.421)	0.534
M-ISI bother score change	0.293 (−0.608, 1.195)	0.524

Abbreviations: BMI, body mass index; AUASS, American Urological Symptom Score; M-ISI, Male Incontinence Symptom Index.
